# Prospective study to characterize adalimumab exposure in pediatric patients with rheumatic diseases

**DOI:** 10.1186/s12969-023-00930-8

**Published:** 2024-01-02

**Authors:** Tatjana Welzel, Klervi Golhen, Andrew Atkinson, Verena Gotta, David Ternant, Jasmin B. Kuemmerle-Deschner, Christine Michler, Gilbert Koch, Johannes N. van den Anker, Marc Pfister, Andreas Woerner

**Affiliations:** 1 Pediatric Pharmacology and Pharmacometrics, University Children’s Hospital Basel, University of Basel, Basel, Switzerland; 2 Division of Pediatric Rheumatology, Department of Pediatrics and Autoinflammation Reference Centre Tuebingen (arcT), University Hospital Tuebingen, Tuebingen, Germany; 3 Pediatric Rheumatology, University Children’s Hospital Basel, University of Basel, Basel, Switzerland; 4 Pediatric Research Center, University Children’s Hospital Basel, University of Basel, Basel, Switzerland; 5 Division of Infectious Diseases, Washington University School of Medicine in St. Louis, St. Louis, MO USA; 6 Université de Tours, service de pharmacologie médicale, Tours France, Université de Tours, EA 4245 T2I, Tours, France

**Keywords:** Pharmacokinetics, Therapeutic drug monitoring, Pharmacodynamics, Target concentration, Drug exposure, Heterogeneity, bDMARDs

## Abstract

**Background:**

In pediatric rheumatic diseases (PRD), adalimumab is dosed using fixed weight-based bands irrespective of methotrexate co-treatment, disease activity (DA) or other factors that might influence adalimumab pharmacokinetics (PK). In rheumatoid arthritis (RA) adalimumab exposure between 2–8 mg/L is associated with clinical response. PRD data on adalimumab is scarce. Therefore, this study aimed to analyze adalimumab PK and its variability in PRD treated with/without methotrexate.

**Methods:**

A two-center prospective study in PRD patients aged 2–18 years treated with adalimumab and methotrexate (G_A-M_) or adalimumab alone (G_A_) for ≥ 12 weeks was performed. Adalimumab concentrations were collected 1–9 (maximum concentration; C_max_), and 10–14 days (minimum concentration; C_min_) during ≥ 12 weeks following adalimumab start. Concentrations were analyzed with enzyme-linked immunosorbent assay (lower limit of quantification: 0.5 mg/L). Log-normalized C_min_ were compared between G_A-M_ and G_A_ using a standard t-test.

**Results:**

Twenty-eight patients (14 per group), diagnosed with juvenile idiopathic arthritis (71.4%), non-infectious uveitis (25%) or chronic recurrent multifocal osteomyelitis (3.6%) completed the study. G_A-M_ included more females (71.4%; G_A_ 35.7%, *p* = 0.13). At first study visit, children in G_A-M_ had a slightly longer exposure to adalimumab (17.8 months [IQR 9.6, 21.6]) compared to G_A_ (15.8 months [IQR 8.5, 30.8], *p* = 0.8). Adalimumab dosing was similar between both groups (median dose 40 mg every 14 days) and observed DA was low. Children in G_A-M_ had a 27% higher median overall exposure compared to G_A_, although median C_min_ adalimumab values were statistically not different (*p* = 0.3). C_min_ values ≥ 8 mg/L (upper limit RA) were more frequently observed in G_A-M_ versus G_A_ (79% versus 64%). Overall, a wide range of C_min_ values was observed in PRD (0.5 to 26 mg/L).

**Conclusion:**

This study revealed a high heterogeneity in adalimumab exposure in PRD. Adalimumab exposure tended to be higher with methotrexate co-treatment compared to adalimumab monotherapy although differences were not statistically significant. Most children showed adalimumab exposure exceeding those reported for RA with clinical response, particularly with methotrexate co-treatment. This highlights the need of further investigations to establish model-based personalized treatment strategies in PRD to avoid under- and overexposure.

**Trial registration:**

NCT04042792, registered 02.08.2019.

**Supplementary Information:**

The online version contains supplementary material available at 10.1186/s12969-023-00930-8.

## Background

Juvenile idiopathic arthritis (JIA) and non-infectious uveitis are common pediatric rheumatic diseases (PRD). Effective treatment of PRD is important to avoid chronic morbidity, diminished health-related quality of life, functional impairment, and long-term sequelae [1–4]. In recent years, treat-to-target (T2T) strategies, consensus treatment plans and treatment recommendations have been established to optimize the care and disease management of PRD patients [5–8]. These steps, together with the availability and approval of biological disease modifying antirheumatic drugs (bDMARDs) for use in pediatric patients, have improved PRD outcomes compared to historical cohorts [9, 10].

For two decades, tumor necrosis factor inhibitors (TNFi) are used in the treatment of PRD. The PRD treatment with TNFi aims to achieve inactive disease by elimination/neutralization of disease-mediating targets. Adalimumab (ADM) is an approved TNFi for the treatment of polyarticular JIA (PJIA), enthesitis associated arthritis (ERA) and non-infectious idiopathic uveitis. Oligoarticular JIA (OJIA) can be treated with ADM if there is inadequate response or intolerance to non-steroidal anti-inflammatory drugs (NSAIDs) and/or intra-articular steroids and at least one conventional disease modifying antirheumatic drugs (cDMARDs) [11]. In PRDs, ADM has been dosed per body surface area (BSA) in the past, and label recommendation recently switched to fixed weight-based dosing regimens with 20 mg subcutaneous (s.c) every other week (EOW) in infants and children weighing 10 to 30 kg, and 40 mg s.c. EOW in those ≥ 30 kg (like adults).

Disease activity (DA), co-treatment with methotrexate (MTX) and individual patients’ characteristics are prone to influence drug exposure and treatment effectiveness. Growing evidence indicates a relationship between TNFi exposure and DA [12, 13]. High DA seems to be associated with lower drug exposure [14]. Low drug exposure can result in treatment ineffectiveness and risk of anti-drug antibody (ADA) development [15, 16]. However, higher exposure than needed for DA control can result in higher risk of adverse events and higher drug costs [17–19]. Furthermore, co-treatment with MTX may increase ADM concentrations [20]. As ADM is often combined with MTX in PRD patients, this might be an additional aspect to be considered during treatment and tapering. According to Krieckaert et al., population-based ADM concentration ranges associated with clinical response in adult patients with rheumatoid arthritis (RA), vary between 2 and 8 mg/L [21]. In adults with rheumatic diseases there is an increasing use of therapeutic drug monitoring (TDM) in clinical practice to provide more individualized drug dosing. However, TDM is difficult to use in PRD patients treated with ADM due to limited pharmacokinetic (PK) and pharmacodynamic (PD) knowledge, highlighting an urgent need of performing such investigations to optimize T2T strategies [22, 23]. The goal of this study was to investigate ADM concentrations in PRD patients treated with ADM, with and without MTX, to (i) better understand ADM exposure and its variability, (ii) analyze concentration changes over time, and (iii) increase current PK knowledge that will facilitate model-based personalized treatment in children with PRD.

## Patients and methods

### Study design

This was an observational two-center prospective pilot-study in PRD patients treated with ADM. Children and adolescents aged 2 to 18 years were eligible if they had a confirmed diagnosis of JIA, non-infectious idiopathic uveitis or chronic recurrent multifocal osteomyelitis (CRMO), and if they were treated ≥ 12 weeks with ADM s.c. with or without MTX (s.c. or orally) between 08/2019 and 12/2021. Exclusion criteria were concomitant treatment with additional bDMARDs or cDMARDs, pregnancy, inability to comply with the study protocol and other concomitant chronic diseases, which could influence drug elimination. After informed consent (IC) process, study participants were subdivided into two study groups (G_A-M,_ G_A_) based on their clinical treatment regimen. Study group G_A-M_ consisted of patients treated with ADM and MTX; study group G_A_ consisted of children receiving ADM monotherapy. ADM s.c. was administered either by the parent, the patient or a nurse at home. Parents/patients were trained by health care professionals at treatment start. For each study participant, ADM maximum concentrations (C_max_) were collected 1 to 9 days and minimum concentrations (C_min_) 10 to 14 days’ post-administration during clinical visits. In addition, naïve children with clinical indication of ADM start were able to participate in a third study group (G_N_) with ADM concentration measurements 3 to 7 days (C_max_, Visit A) and 10 to 14 days (C_min_, Visit B) after their first ADM administration. A detailed study schedule is presented in Supplementary material S1. Study data was captured in the web-based electronically secured database secuTrial®. The study was conducted in accordance with the Declaration of Helsinki, Good Clinical Practice, the Human Research Act, and the Human Research Ordinance. The study was approved by the ethics committees of Nordwest- und Zentralschweiz (EKNZ, 2019–00916) and medical faculty and University Hospital Tuebingen (321/2019B01). The study was registered at ClinicalTrials.gov (NCT04042792).

### Data collection

Baseline characteristics and treatment information (e.g. dose and administration frequency of ADM/MTX, concomitant treatment categorized as NSAIDs, systemic corticosteroids and intra-ocular steroids) were collected. Intra-articular steroid application was captured if administered between first and last study visit. Furthermore, DA scores and clinical and laboratory routine data were assessed at each study visit. Routinely measured laboratory parameters included C-reactive protein (CRP) and/or erythrocyte sedimentation rate (ESR). In addition, clinical routine data available in patient health records were captured from ADM treatment start until study inclusion.

### Disease Activity (DA) assessment

DA was captured by the physician global assessment (PGA) and patient’s/parents’ global assessment (PPGA). PGA and PPGA were recorded on a 10 cm visual analog scale (VAS) with 0 representing no DA and 10 representing maximum DA. In addition, DA was captured for patients with JIA by the Juvenile Arthritis Disease Activity Score (JADAS)-10. JADAS cut-off values for the DA status were based on Trincianti et al. [24] as follows: 1) OJIA: inactive DA ≤ 1.4; minimal DA 1.5–4; moderate DA 4.1–13; high DA > 13; 2) PJIA: inactive DA ≤ 2.7; minimal DA 2.8–6; moderate DA 6.1–17; high DA > 17. In ERA patients, cut-off values were based on active joint count (1–4 active joints: OJIA cut-off, ≥ 5 active joints: PJIA cut-off). Uveitis was defined by cells in the field of the anterior chamber in line with the standardization of the uveitis nomenclature (SUN) (grade 0: < 1 cell, grade 0.5 + : 1–5 cells; grade 1 + : 6–15 cells; grade 2 + : 16–25 cells; grade 3 + : 26–50 cells; grade 4 + : > 50 cells) [25]. The SUN grades were translated to DA status as following: inactive: grade 0; minimal: grade 0.5 + ; mild: grade 1 + ; moderate: 2 + and severe: grade ≥ 3 + [26].

### Adalimumab concentration measurement

Sample management was standardized (Supplemental material S2). Aliquots were transferred on dry ice by batches to the accredited collaborating laboratory (MVZ Dr Eberhard & Partner Dortmund, Dortmund, Germany). Analyses were performed with the same charge of an enzyme-linked accredited immunosorbent assay (EIA) using Dynex DSX automated ELISA system. Assay detection limit was 0.1 mg/L and lower and upper limits of quantification were 0.5 and 12 mg/L, respectively. Sera exceeding the upper limit of quantification were diluted 1:4, thus creating an upper limit of 48 mg/L.

### Primary and secondary outcomes

The primary outcome was to compare ADM C_min_ ≥ 12 weeks after first ADM administration in study participants with (G_A-M_) and without MTX (G_A_). Secondary outcomes included comparison of ADM concentrations and DA during treatment, dosing regimen, and the influence of body weight. Post hoc analysis included investigation of ADM C_max_ and C_min_ in treatment naïve patients (G_N_) as well as PGA, PPGA and laboratory parameters since ADM start.

### Hypothesis and sample size calculation

It was hypothesized that children in G_A-M_ have higher ADM C_min_ compared to G_A_. To determine if this difference was statistically significant, mean concentrations were estimated (following appropriate transformations to achieve normality) for each group (details in the protocol). With 90% power and a two-sided statistical significance level of 5%, recruitment was estimated at 2 × 12 = 24 patients (altogether) to detect a difference between the groups of 1.4 standard deviations or more. Furthermore, a 10% drop out in each group was assumed, resulting a recruitment target of 2 × 14 = 28 children for this study.

### Statistical analysis

Patient characteristics were summarized using descriptive statistics; missing data addressing routine parameters are marked. Categorical values were represented as number (%) and continuous values as median (interquartile ranges; IQR). Comparative analyses between G_A-M_ and G_A_ were conducted using the chi-square test for categorical variables and the Wilcoxon test for continuous variables. As stated above, the primary analysis compared log transformed mean C_min_ using a standard t-test. Linear regression models were fitted to determine associations between the individual (log transformed) adalimumab concentrations and other factors (such as study group, visit age and gender) that may influence drug exposure as independent variables. Univariable and multivariable linear mixed effect models investigating relationship between adalimumab concentrations (log-transformed) and study group, age at visit were fitted with a random intercept slope for each participant. All analysis and graphs were performed utilizing R version 4.2.2.

## Results

### Study population

**Fig. 1:**
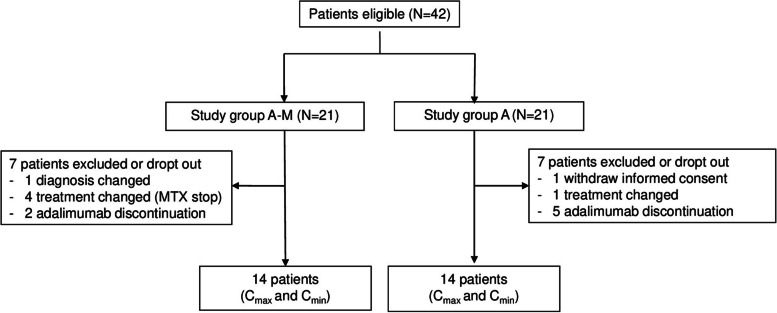
Study Flow chart. Abbreviation: *Study group A-M* adalimumab and methotrexate co-treatment, *Study group A* adalimumab alone, *C*_*max*_ maximum adalimumab concentration collected 1 to 9 days after adalimumab administration, *C*_*min*_ minimum adalimumab concentration collected 10 to 14 days after adalimumab administration, *MTX* methotrexate

In total, 14 patients in G_A-M_ and G_A_ completed the study between September 26, 2019 and June 28, 2021 (Fig. 1). G_A-M_ included more females (71.4%) and children tended to be diagnosed at an earlier median age (6.3 years [IQR 2.4, 9.0]) compared to G_A_ (35.7% females, 8.8 years [IQR 5.7, 10.1], *p* = 0.2). At first study visit, median age was similar in G_A-M_ and G_A_. Most children (*n* = 20, 71.4%) were diagnosed with JIA; a history/active JIA-associated uveitis (JIA-uveitis) was documented in six of 12 children with JIA in G_A-M,_ and in three out of eight in G_A_ (Table 1, Supplementary material S3). Seven (25%) children were diagnosed as non-infectious idiopathic uveitis with five being included in G_A_. One patient in G_A_ was diagnosed with CRMO. At time of study inclusion, children in G_A-M_ were treated slightly longer with ADM (17.8 months [IQR 9.6, 21.6]) compared to those in G_A_ (15.8 months [IQR 8.5, 30.8], *p* = 0.8). Median ADM dose and administration frequency were similar between both groups. Children in G_A-M_ received weekly MTX (median dose per BSA 9.0 [6.6, 9.8] mg/m^2^). Concomitant PRD treatment included systemic corticosteroids, NSAIDs and ocular steroids (Table 1, Supplementary material S4). Median follow-up time between first and last study visit was 4.4 months [IQR 3.5, 6.2] and 2.9 months [IQR 2.6, 3.4] for G_A-M_ and G_A_, respectively (Table 1).

**Table 1:** Characteristics of children with PRD treated with adalimumab ≥ 12 weeks with or without methotrexate

	**Total** **(*N* = 28)**	**Study group A-M** **(G**_**A-M**_**, *n* = 14)**	**Study group A** **(G**_**A**_**, *n* = 14)**	***p*-value**
**General information study cohort**				
Female sex, n (%)	15 (53.6)	10 (71.4)	5 (35.7)	0.13
Median age at diagnosis, years [IQR]	7.1 [4.4, 10.1]	6.3 [2.4, 9.00]	8.8 [5.7, 10.1]	0.19
Median age at first visit, years [IQR]	11.3 [8.9, 13.2]	11.3 [8.9, 13.5]	11.5 [9.3, 12.8]	0.82
Median body weight, kg [IQR]	38.7 [31.9, 54.1]	35.7 [31.7, 52.7]	43.7 [33.0, 54.9]	0.59
Comorbidities, n (%)	3 (10.7)	0	3 (21.4%)^a^	0.22
**Diagnosis, n (%)**				
JIA	20 (71.4)	12 (85.7)	8 (57.1)	0.21
Idiopathic Uveitis	7 (25.0)	2 (14.3)	5 (35.7)	
CRMO	1 (3.6)	0	1 (7.1)	
**Disease activity**				
Median CRP, mg/L [IQR] (ref < 10) *Missing, n (%)*	0.3 [0.1, 1.1] *1 (3.6)*	0.1 [0.1, 0.3]	0.6 [0.3, 1.3] *1 (7.1)*	0.01
Median ESR, mm/h [IQR] (ref < 15) *Missing, n (%)*	5.5 [4.0, 6.3] *4 (14.3)*	6.0 [5.0, 7.0] *1 (7.1)*	4.00 [4.0, 6.0] *3 (21.4)*	0.28
Median PGA, cm [IQR]	0 [0, 1.0]	0.50 [0, 1.8]	0 [0, 1.0]	0.53
Median PPGA, cm [IQR]	0 [0, 1.0]	1.00 [0, 1.8]	0 [0, 0]	0.05
**Adalimumab treatment**				
Median time since start, months [IQR]	17.6 [8.4, 25.1]	17.8 [9.6, 21.6]	15.8 [8.5, 30.8]	0.78
Absolute median dose, mg [IQR]	40.0 [28.8, 40.0]	40.0 [25.0, 40.0]	40.0 [30.0, 40.0]	0.77
Median dose per BSA, mg/m^2^ [IQR]	25.3 [22.9, 28.3]	25.1 [21.3, 32.0]	25.4 [23.6, 27.2]	0.93
Median frequency, days [IQR]	14.0 [14.0, 14.0]	14.0 [14.0, 14.0]	14.0 [14.0, 14.0]	0.35
**Methotrexate treatment**				
Median time since start, months [IQR]	n.a	23.4 [19.7, 59.2]	n.a	n.a
Absolute median dose, mg [IQR]		11.0 [10.0, 15.0]		
Median dose per BSA, mg/m^2^ [IQR]		9.0 [6.6, 9.8]		
Median frequency, days [IQR]		7.0 [7.0, 7.0]		
**Concomitant treatment at inclusion, n (%)**				
Corticosteroids, systemic^b^	2 (7.1)^b^	2 (14.3)^b^	0	0.09
NSAIDs, on demand	4 (14.3)	2 (14.3)	2 (14.3)	
NSAIDs, fix administration	3 (10.7)	1 (7.1)	2 (14.3)	
Ocular steroids^b^	4 (14.3)	4 (28.6)	0	

Abbreviation: *BSA* Body surface area, *CRMO* chronic recurrent multifocal osteomyelitis, IQR Inter-quartile ranges, *i.v.* intravenous*, JIA* juvenile idiopathic arthritis, *kg* kilogram, *mg* milligram, *n.a.* not available, *NSAIDs* non-steroidal anti-inflammatory drugs, *p.o* per os, GA-M study group adalimumab and methotrexate, GA study group adalimumab, p—values: chi-square test for categorical variables; Wilcoxon test for continuous variables

^a^Comorbidities: patella luxation left (*n* = 1), autism spectrum disorder (*n* = 1), muscular tension (*n* = 1)

^b^dosing regimen is shown in the Supplementary material S4

### Primary outcome

**Fig. 2:**
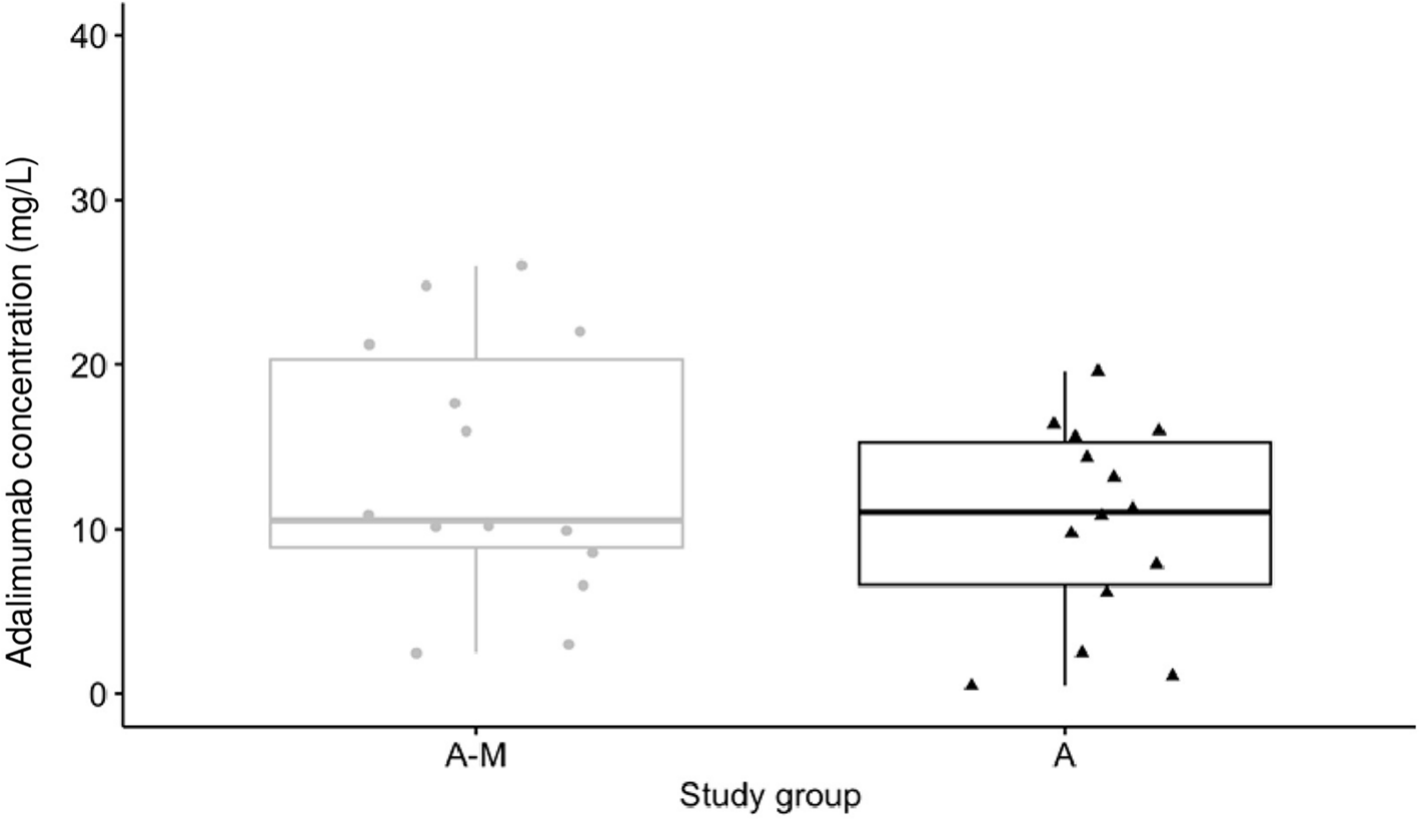
Boxplot of adalimumab minimal concentrations (C_min_) in children with PRD with and without methotrexate co-treatment. Legend: Adalimumab C_min_ concentrations collected 10–14 days after adalimumab administration in pediatric patients with rheumatic diseases in study group A-M (adalimumab and methotrexate treatment) and study group A (adalimumab alone); t-test of log transformed C_min_ *p* = 0.3. The box of the boxplot limits the interquartile ranges (IQR)

Median ADM C_min_ was 10.6 mg/L [IQR 8.9, 20.3] in G_A-M_ compared to 11.1 mg/L [IQR 6.6, 15.3] in G_A_, but this difference was not statistically significant at the 5% level (t-test of log transformed C_min_ measurements; *p* = 0.3, Table 2, Fig. 2).

**Table 2:** Adalimumab concentration and treatment information in children with PRD

	**Study group A-M** **(G**_**A-M**_**, *n* = 14)**		**Study group A** **(G**_**A**_**, *n* = 14)**		**Total** **(*n* = 28)**	
	C_max_	C_min_	C_max_	C_min_	C_max_	C_min_
**Adalimumab concentration, mg/L**						
Median [IQR]	16.6 [11.9, 25.0]	10.6 [8.9, 20.3]	16.0 [8.2, 18.7]	11.1 [6.6, 15.3]	16.0 [10.6, 22.3]	10.9 [7.6, 16.1]
Median overall [IQR]	15.6 [10.1, 22.3]		12.3 [7.4, 16.6]		13.8 [8.4, 18.5]	
Mean (SD)	17.5 (7.5)	13.5 (7.8)	14.2 (7.2)	10.4 (6.0)	15.8 (7.4)	12.0 (7.0)
Range (min, max)	5.2, 28.4	2.5, 26.0	4.2, 25.2	0.5, 19.6	4.2, 28.4	0.5, 26.0
≥ 8 mg/L, patients (%)	12 (85.7)	11 (78.6)	10 (71.4)	9 (64.3)	22 (78.6)	20 (71.4)
**Time after last adalimumab administration, days**						
Median [IQR]	3.5 [2.0, 6.8]	12.0 [10.3, 13.0]	5.0 [4.3, 6.8]	12.0 [11.0, 13.0]	5.0 [2.0, 7.0]	12.0 [11.0, 13.0]
Range (min, max)	1.0, 7.0	10.0, 14.0	2.0, 8.0	10.0, 14.0	1.0, 8.0	10.0, 14.0
**Time to adalimumab sample collection after adalimumab start, months**						
Median [IQR]	11.2 [7.50, 17.4]	18.2 [9.5, 22.0]	11.8 [7.4, 27.8]	16.2 [8.3, 31.1]	11.8 [7.3, 22.3]	17.9 [8.2, 25.6]
Range (min, max)	3.5, 62.3	4.8, 66.7	3.6, 44.4	3.7, 41.5	3.5, 62.3	3.7, 66.7
**Adalimumab dose administered**						
Absolute median dose, mg [IQR]	40.0 [25.0, 40.0]		40.0 [30.0, 40.0]		40.0 [28.8, 40.0]	
Median dose per BSA, mg/m^2^ [IQR]	25.1 [21.3, 31.2]	24.5 [21.0, 32.0]	25.1 [23.6, 26.8]	25.4 [23.8, 27.0]	25.1 [22.9, 27.6]	25.1 [22.8, 28.6]

Abbreviation *BSA* Body surface area, *IQR* Interquartile ranges*, SD* standard deviation*, mg* milligram, mL milliliter, *min* minimum, *max* maximum, *m* meter, *G*_*A-M*_ study group adalimumab and methotrexate, *G*_*A*_ study group adalimumab

### Secondary outcomes

Most children with JIA had inactive or minimal DA classified by JADAS-10 at first study visit (G_A-M_ 66.7%; G_A_ 87.5%) with stable DA or slight DA improvement observed at last study visit (G_A-M_ 75%; G_A_ 87.5%). DA of uveitis in children with JIA or idiopathic uveitis was captured as absent, minimal, or mild at first and last study visit with only small changes between both visits. Median CRP and median ESR were normal in G_A-M_ and G_A_ at both study visits (Table 3, Supplementary material figure S1A and S1B). In addition, median PGA and median PPGA were overall low (Table 3, Supplementary material S5). Uni- and multivariable linear mixed effect models revealed no statistically significant relationship between ADM concentrations (log-transformed) and study group and age at visit (Supplementary material S6).

**Table 3:** Disease activity at first and last study visit in children with PRD

	**Study group A-M (G**_**A-M**_**)**		**Study group A (G**_**A**_**)**	
	**First study visit**	**Last study visit**	**First study visit**	**Last study visit**
**Disease activity parameters, median [IQR]**				
CRP, mg/L (ref < 10) *Missing, n (%)*	0.1 [0.1, 0.3]	0.300 [0.1, 2.1]	0.6 [0.3, 1.3] *1 (7.1)*	0.5 [0.3 1.0] *1 (7.1)*
ESR, mm/h (ref < 15) *Missing, n (%)*	6.0 [5.0, 7.0] *1 (7.1)*	5.0 [5.0, 7.0] *1 (7.1)*	4.0 [4.0, 6.0] *3 (21.4)*	7.0 [5.0, 8.0] *3 (21.4)*
PGA, cm	0.5 [0, 1.8]	1.0 [0, 1.8]	0 [0, 1.0]	0 [0, 0]
PPGA, cm	1.0 [0, 1.8]	0.5 [0, 2.0]	0 [0, 0]	0 [0, 0]
**Disease activity in children with JIA (based on the JADAS-10), n (%)**				
	*n* = 12		*n* = 8	
Inactive	4 (33.3)	4 (33.3)	6 (75.0)	7 (87.5)
Minimal	4 (33.3)	5 (41.7)	1 (12.5)	0
Moderate	2 (16.7)	3 (25.0)	1 (12.5)	1 (12.5)
High	1 (8.3)	0	0	0
Missing	1 (8.3)	0	0	0
**Disease activity in children with uveitis (based on cells in field by SUN), n (%)**				
	*n* = 8 *(n* = *2 idiopathic uveitis, n* = *6 JIA-uveitis)*		*n* = 8 *(n* = *5 idiopathic uveitis, n* = *3 JIA-uveitis)*	
Absent	8 (100)	5 (62.5)	5 (62.5)	7 (87.5)
Minimal	0	2 (25.0)	2 (25.0)	0
Mild	0	0	1 (12.5)	1 (12.5)
Missing	0	1 (12.5)	0	0

Abbreviation *CRP* c-reactive protein, *cm* centimetre, *ESR* erythrocyte sedimentation rate, *JIA* Juvenile idiopathic arthritis, *JADAS* Juvenile Arthritis Disease Activity Score, *IQR* Inter-quartile ranges*, L* litre, *mg* milligram, *PGA* physician global assessment, *PPGA* patient’s /parents global assessment, *ref*. reference values, *SUN* standardization of the uveitis nomenclature*, G*_*A-M*_ study group adalimumab and methotrexate, *G*_*A*_ study group adalimumab

**Fig. 3:**
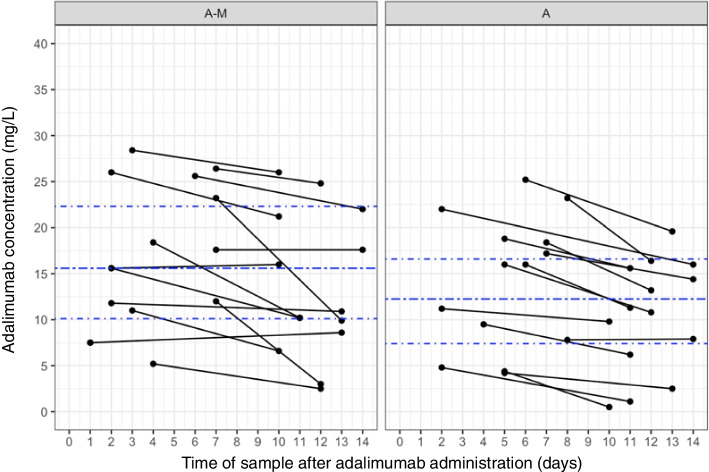
Adalimumab exposure in children with PRD with and without methotrexate co-treatment depending on sample time. Legend: Adalimumab exposure in children with PRD treated with adalimumab and methotrexate (A-M) and adalimumab alone (A) after ≥ 12 weeks. Maximal adalimumab concentrations were collected after 1 to 9 days (C_max_) and minimal concentrations (C_min_) after 10 to 14 days. The *dash blue lines* represent the interquartile ranges [IQR] and the median concentrations per study group (A-M: 15.6 mg/L [IQR 10.1, 22.3]; A: 12.3 mg/L [IQR 7.4, 16.6])

**Fig. 4:**
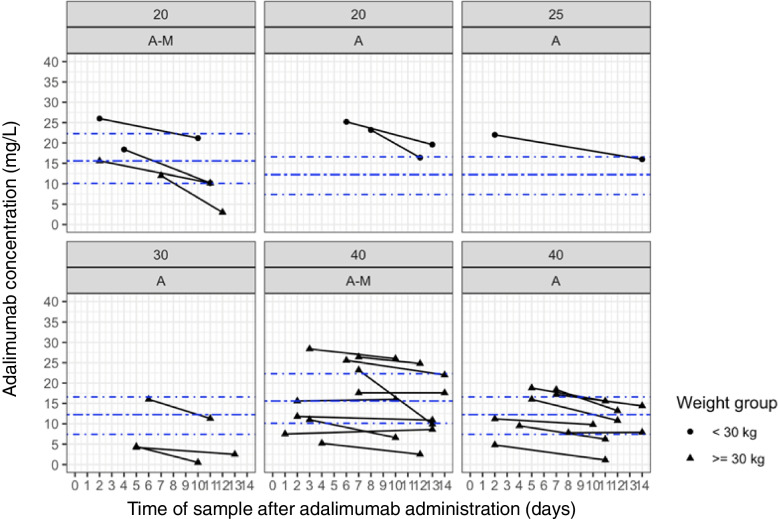
Adalimumab exposure in children with PRD with and without methotrexate co-treatment depending on dosing. Legend: Adalimumab exposure in children with PRD treated with adalimumab and methotrexate (A-M) or adalimumab alone (A) ≥ 12 weeks with adalimumab absolute doses of 20, 25, 30 or 40 mg. Maximum adalimumab concentrations were collected after 1 to 9 days (C_max_) and minimum concentrations after 10 to 14 days (C_min_). The *dash blue lines* represent the interquartile ranges [IQR] and the median concentrations per study group (A-M: 15.6 mg/L [IQR 10.1, 22.3]; A: 12.3 mg/L [IQR 7.4, 16.6]). *Triangle*: body weight ≥ 30 kg, *dot*: body weight < 30 kg

Most children treated ≥ 12 weeks showed higher ADM C_max_ compared to C_min_ values (Fig. 3). Furthermore, a high inter-individual variability in C_min_ was detected (0.5 to 26 mg/L) with C_min_ ranges between 2.5 to 26 mg/L and 0.5 to 19.6 mg/L in G_A-M_ and G_A_, respectively. In this study, C_min_ of 78.6% (*n* = 11) of children in the G_A-M_ and 64.3% (*n* = 9) of children in the G_A_ group were ≥ 8 mg/L (Table 2, Fig. 3). In G_A-M_, all children were treated with a fixed ADM dosing regimen (*n* = 4 with 20 mg, *n* = 10 with 40 mg) and in G_A_ ten children (*n* = 2 with 20 mg, *n* = 8 with 40 mg). Four children in G_A_ received BSA-adjusted dosing (24 mg/m^2^) resulting in 25 mg ADM injection in one and 30 mg in three (Fig. 4). The median ADM overall exposure (C_min_ and C_max_) was 15.6 mg/L [IQR 10.1, 22.3] in G_A-M_ and 12.3 mg/L [IQR 7.4, 16.6] in G_A_. The distribution of ADM concentrations for the dosing regimen are shown in Fig. 4, showing that concentrations are well scattered around IQR, independent of dosing and dosing approach (fixed dosing versus BSA-based dosing). When analyzing all 56 measured ADM concentrations, the median overall concentration was 13.8 mg/L [IQR 8.4, 18.5]. Of five children weighting < 30 kg, four had a fixed dosing regimen. The 23 children weighting > 30 kg received either a fixed ADM dosing regimen or a BSA-adjusted regimen; no child in the G_A_ group had adalimumab C_min_ > 18.5 mg/L (Fig. 4). No influence of systemic corticosteroids or ocular steroids on ADM concentrations could be shown (Supplementary material figure S3). During clinical routine monitoring, ADA assessment was performed in 10 children of the whole cohort. No ADA were detected.

### Additional post hoc exploratory analyses

The eight ADM naïve patients (G_N_) consisted of six females and two males with a median age of 12.8 years [IQR 11.0, 15.6] at study visit. They were diagnosed with JIA (62.5%) and idiopathic uveitis (37.5%). All children received 40 mg ADM, five had MTX co-treatment. After first ADM injection, the median ADM C_min_ was 5.8 mg/L [IQR 3.8, 7.3] (Supplementary material S7). Assessed DA over the treatment since ADM start showed a decrease of PPGA and PGA particularly in the first year in both study groups. In G_A-M_, the PGA and PPGA tended to plateau at a 1 cm, whereas a secondary increase was detected in G_A_ until first study visit (Supplementary material figure S2A and B).

## Discussion

This prospective pilot-study increases understanding of ADM PK and its variability in children with PRD with and without MTX co-treatment. Children with MTX co-treatment (G_A-M_) had a 27% higher median overall exposure compared to ADM monotherapy (G_A_), although median ADM C_min_ were not statistically different between both groups. C_min_ values ≥ 8 mg/L were more frequent observed in G_A-M_ versus G_A_ (78.6% versus 64.3%). A high variability in ADM concentrations were detected in both study groups. In this study cohort the overall DA was low in both groups, PGA and PPGA of patients in the G_A-M_ group tended to plateau. These findings indicate and strengthen the need for personalized dosing strategies to optimize treatment in children with PRD.

In this study, children with PRD showed high inter-individual variability in ADM exposure, which tended to be increased by MTX co-treatment. The ADM concentrations in most children in this study exceeded concentration ranges reported in adults with RA and other inflammatory rheumatic diseases. In adult patients with RA treated with ADM 40 mg EOW ≥ 12 to 28 weeks, trough concentrations (C_trough_) ranging from 4.4 to 8 mg/L have been reported to be sufficient to reach adequate clinical response [12, 27]. In addition, C_trough_ cut-off values of 1.3 mg/L at 6 months and of 1.0 mg/L at 12 months of treatment were associated with a good DA control in RA patients [28]. ADM concentrations > 8 mg/L were shown to have no additional beneficial effect on DA in RA [12]. Chen et al. suggested ADM dose reduction in remitted RA patients with C_trough_ > 6.4 mg/L [29]. Population-based ADM concentration ranges associated with clinical response have been described for RA to vary between 2 to 8 mg/L [21]. Further data for spondylarthritis and psoriatic arthritis as well as for other inflammatory diseases, e.g. inflammatory bowel disease or psoriasis indicate ADM target concentrations during maintenance therapy between 1 to 10 mg/L [21, 30–34]. Due to the quite consistent results in different inflammatory conditions, it seems reasonable to extrapolate these target ranges for PRD. In this study, 78.6% of children treated with ADM and MTX and 64.3% of children with ADM monotherapy had C_min_ ≥ 8 mg/L. The observation towards higher ADM exposure in children compared to adults with RA or other inflammatory diseases is in line with existing studies and raises the question if children with PRD and remission might have higher ADM exposure as needed for DA control. Children with PJIA aged 4 to 17 years treated with ADM 40 mg EOW and MTX had mean C_trough_ of 10.4 mg/L (*n* = 14) at week 16 and 14.4 mg/L at week 60 [35]. PJIA children (*n* = 6) treated with 20 mg ADM EOW and MTX had a mean C_trough_ of 6.73 mg/L after 16 weeks with increase to 14.3 mg/L at week 60 [35]. Doeleman et al. reported median C_trough_ of 14.9 mg/L [IQR 10.3, 16.2] in children with JIA, who had adequate response to ADM 24 mg/m^2^ (maximum 40 mg) EOW [13]. Rashid et al. detected median ADM C_trough_ of 10.2 mg/L in children with JIA [22]. In children with ERA treated with ADM 24 mg/m^2^ (maximum 40 mg) EOW, mean C_trough_ were 7.5 to 11.8 mg/L between weeks 12 and 52 [20]. Mean ADM C_trough_ in children with PJIA aged 2 to 4 years or age ≥ 4 years weighing < 15 kg treated with ADM 24 mg/m^2^ (maximum 20 mg) EOW were comparable to those measured in children aged 4 to 17 years [35, 36]. In this study, we assessed C_min_ and C_max_ ADM concentrations, whether other studies used C_trough_. To date, it is unclear whether sampling at C_trough_ compared to other time points (e.g. C_max_) is important [21]. In RA patients, one study observed correlations between time of ADM administration, sampling time, and concentration, while another study did not show any influence of sample timing, with the exception of C_trough_ predicting successful dose reduction [37, 38]. In our study, higher C_max_ compared to C_min_ concentrations strengthen the importance of sampling time, even in long-term treatment. The high inter-individual variability, the ADM exposure exceeding those of other inflammatory conditions, and the possible importance of sampling time highlight the need of better PK understanding in PRD patients to optimize dosing regimen.

Although a trend to highest exposure under MTX co-treatment was observed, no statistical difference in ADM C_min_ for ADM monotherapy or MTX co-treatment was documented. Existing data suggest an influence of treatment response on ADM exposure. ADM concentrations have been described to be lower in patients with high DA or treatment failure compared to those in remission or with low DA [13, 39–42]. This could be explained by the lower amount of TNF targets in inactive disease resulting in higher ADM exposure with standard dosing than needed for TNF target neutralization [43]. Most children had inactive or minimal DA and have been treated long-term. The overall good DA control might be a possible explanation for the relatively high exposure in this study, raising the question whether the exposure was higher than needed to control DA. This would, strengthen a taper approach after stable remission is achieved. Particularly, as higher ADM exposure in remitted patients is not associated with additional beneficial effects on DA but increased risk of adverse events and higher drug costs [12, 17–19, 27]. However, low exposure may increase the risk for ADA, associated with loss of treatment response [16, 44–46]. The risk of ADA seems to increase over time, whereas MTX co-treatment might reduce the risk [44, 47, 48]. In this study, no standardized ADA assessment was performed. However, in the ten assessed children no ADA were detected. This contrasts with other studies, although comparisons are difficult due to differences in assays [23]. Up to date, no consistent observation that ADA formation is associated with secondary ADA treatment failure has been shown [23] and, there is evidence that ADM with and without MTX co-treatment is effective in long-term-treatment with comparable treatment responses [49, 50]. This highlights the importance of defining concentration target ranges, PK driven personalized treatment approaches and taper strategies in PRD with sustained remission to avoid ADM over- and underexposure.

This study has several limitations. First, the C_min_ difference between G_A-M_ and G_A_ was smaller than expected and the inter-individual variability relatively large, and hence the sample size of 28 patients may have been too small to demonstrate significant C_min_ differences. However, PK studies in pediatrics can be designed with six to 12 subjects [51], and we could gain valuable insights in ADM exposure and ADM PK in children with PRD. Second, based on study design, children with stable remission and therefore clinical indication of MTX/ADM discontinuation were replaced, which might be associated with a certain selection bias. As non-adherence or unsteady administration can be a potential confounder by studying drug exposure [42], this study design ensured high quality data of compliant patients. Third, in this pilot-study we focused on ADM exposure in children with PRD and long-term treatment and data was mainly originated from school age children and adolescents. Low ADM concentrations were observed infrequently, what might be explained as no infants participated, C_min_ instead of C_trough_ concentrations were collected and participants had long-term treatment with overall good DA control. As children with lower age and weight might tend to lower bDMARD exposure, their ADM concentrations might be lower [23]. Further research in early disease stages, infants and high disease activity is needed. Fourth, a heterogeneous PRD population (JIA, idiopathic uveitis, CRMO) was included although, most children were JIA patients.

## Conclusion

This prospective pilot-study in children with PRD and long-term ADM treatment with and without MTX co-treatment aimed to analyze ADM PK and its variability to better understand ADM exposure in children with PRD. Children with MTX co-treatment (G_A-M_) had a 27% higher median overall exposure compared to ADM monotherapy (G_A_), although C_min_ were not statistically significant different between both groups. A high overall variability in C_min_ was observed in both groups, and most children with PRD had ADM C_min_ exceeding upper target ranges reported for RA (≥ 8 mg/L) and other inflammatory diseases, particularly those with MTX co-treatment (78.6% versus 64.3%). These findings, together with target ADM concentration ranges based on exposure-clinical response relationships, highlights the need of further pharmacological investigation to establish model-based personalized treatment approaches to avoid particularly drug overexposure in children with PRD.

### Supplementary Information

**Additional file 1:** **Table S1.** Study schedule. **Table S2.** Sample management. **Table S3.** JIA subgroups by study group. **Table S4** Corticosteroids treatment details at inclusion. **Table S5.** Grading by cells in the field of the chamber (SUN) and JADAS-10 scores in PRD patients with juvenile idiopathic arthritis and uveitis. **Table S6.** Univariable and multivariable linear mixed effect models investigating relationship between adalimumab concentrations (log-transformed) and study group, visit age and gender. **Table S7.** Characteristics adalimumab naïve children receiving the first adalimumab dose. **Figure S1.** Adalimumab concentrations and inflammatory marker. **Figure S2.** Adalimumab concentrations and disease activity captured by PGA and PPGA. **Figure S3.** Adalimumab exposure in children with PRD with adalimumab by study group with and without concomitant corticosteroid treatment.

## Data Availability

The data analyzed during the current study are not publicly available due to ethical considerations (no informed consent for further use). In case of reasonable requests, the corresponding author can be contacted, and ethics committee approval might be obtained for further use.

## Abbreviations

ADA: Anti-drug antibodies
ADM: Adalimumab
bDMARD: Biological diseases modifying antirheumatic drugs
BSA: Body surface area
cDMARD: Conventional diseases modifying antirheumatic drugs
C_min_: Minimal concentration (collected 10 to 14 days after last adalimumab administration)
C_max_: Maximal concentration (collected 1 to 9 days after last adalimumab administration)
CRP: C-reactive protein
CRMO: Chronic recurrent multifocal osteomyelitis
DA: Disease activity
EIA: Enzyme-linked immunosorbent assay
EOW: Every other week
ERA: Enthesitis-associated juvenile idiopathic arthritis
ESR: Erythrocyte sedimentation rate
G: Group
IQR: Interquartile ranges
JADAS: Juvenile Arthritis Disease Activity Score
JIA: Juvenile idiopathic arthritis
mg: Milligram
mL: Milliliter
MTX: Methotrexate
NSAIDs: Non-steroidal anti-inflammatory drugs
OJIA: Oligoarticular juvenile idiopathic arthritis
PD: Pharmacodynamics
PK: Pharmacokinetics
PGA: Physician global assessment
PJIA: Polyarticular juvenile idiopathic arthritis
PPGA: Patients/parents global assessment
PRD: Pediatric rheumatic diseases
RA: Rheumatoid Arthritis
S.C.: Subcutaneous
SD: Standard deviation
SUN: Standardization of the uveitis nomenclature
TDM: Therapeutic drug monitoring
T2T: Treat-to-target
TNF: Tumor necrosis factor
TNFi: Tumor necrosis factor inhibitor
VAS: Visual analog scale
μg: Microgram

## References

[1] d'Angelo DM, Di Donato G, Breda L, Chiarelli F (2021). Growth and puberty in children with juvenile idiopathic arthritis. Pediatr Rheumatol Online J.

[2] Moorthy LN, Peterson MG, Hassett AL, Lehman TJ (2010). Burden of childhood-onset arthritis. Pediatr Rheumatol Online J.

[3] Oen K, Tian J, Loughin TM, Shiff NJ, Tucker LB, Huber AM (2021). Causal pathways to health-related quality of life in children with juvenile idiopathic arthritis: results from the ReACCh-Out cohort. Rheumatology (Oxford).

[4] Sen ES, Morgan MJ, MacLeod R, Strike H, Hinchcliffe A, Dick AD (2017). Cross sectional, qualitative thematic analysis of patient perspectives of disease impact in juvenile idiopathic arthritis-associated uveitis. Pediatr Rheumatol Online J.

[5] Ravelli A, Consolaro A, Horneff G, Laxer RM, Lovell DJ, Wulffraat NM (2018). Treating juvenile idiopathic arthritis to target: recommendations of an international task force. Ann Rheum Dis.

[6] Horneff G, Klein A, Ganser G, Sailer-Hock M, Gunther A, Foeldvari I (2017). Protocols on classification, monitoring and therapy in children's rheumatology (PRO-KIND): results of the working group Polyarticular juvenile idiopathic arthritis. Pediatr Rheumatol Online J.

[7] Angeles-Han ST, Ringold S, Beukelman T, Lovell D, Cuello CA, Becker ML (2019). 2019 American College of Rheumatology/Arthritis Foundation Guideline for the Screening, Monitoring, and Treatment of Juvenile Idiopathic Arthritis-Associated Uveitis. Arthritis Care Res (Hoboken).

[8] Constantin T, Foeldvari I, Anton J, de Boer J, Czitrom-Guillaume S, Edelsten C (2018). Consensus-based recommendations for the management of uveitis associated with juvenile idiopathic arthritis: the SHARE initiative. Ann Rheum Dis.

[9] Chhabra A, Robinson C, Houghton K, Cabral DA, Morishita K, Tucker LB (2020). Long-term outcomes and disease course of children with juvenile idiopathic arthritis in the ReACCh-Out cohort: a two-centre experience. Rheumatology (Oxford).

[10] Maccora I, Sen ES, Ramanan AV (2020). Update on noninfectious uveitis in children and its treatment. Curr Opin Rheumatol.

[11] Onel KB, Horton DB, Lovell DJ, Shenoi S, Cuello CA, Angeles-Han ST (2022). 2021 American College of Rheumatology Guideline for the Treatment of Juvenile Idiopathic Arthritis: Therapeutic Approaches for Oligoarthritis, Temporomandibular Joint Arthritis, and Systemic Juvenile Idiopathic Arthritis. Arthritis Rheumatol.

[12] Pouw MF, Krieckaert CL, Nurmohamed MT, van der Kleij D, Aarden L, Rispens T (2015). Key findings towards optimising adalimumab treatment: the concentration-effect curve. Ann Rheum Dis.

[13] Doeleman MJH, de Roock S, El Amrani M, van Maarseveen EM, Wulffraat NM, Swart JF (2021). Association of adalimumab trough concentrations and treatment response in patients with juvenile idiopathic arthritis. Rheumatology (Oxford).

[14] Ternant D, Azzopardi N, Raoul W, Bejan-Angoulvant T, Paintaud G (2019). Influence of Antigen Mass on the Pharmacokinetics of Therapeutic Antibodies in Humans. Clin Pharmacokinet.

[15] Moots RJ, Xavier RM, Mok CC, Rahman MU, Tsai WC, Al-Maini MH (2017). The impact of anti-drug antibodies on drug concentrations and clinical outcomes in rheumatoid arthritis patients treated with adalimumab, etanercept, or infliximab: Results from a multinational, real-world clinical practice, non-interventional study. PLoS One.

[16] Bartelds GM, Krieckaert CL, Nurmohamed MT, van Schouwenburg PA, Lems WF, Twisk JW (2011). Development of antidrug antibodies against adalimumab and association with disease activity and treatment failure during long-term follow-up. JAMA.

[17] Nestorov I (2005). Clinical pharmacokinetics of tumor necrosis factor antagonists. J Rheumatol Suppl.

[18] l'Ami MJ, Krieckaert CL, Nurmohamed MT, van Vollenhoven RF, Rispens T, Boers M (2018). Successful reduction of overexposure in patients with rheumatoid arthritis with high serum adalimumab concentrations: an open-label, non-inferiority, randomised clinical trial. Ann Rheum Dis..

[19] Krieckaert CL, Nair SC, Nurmohamed MT, van Dongen CJ, Lems WF, Lafeber FP (2015). Personalised treatment using serum drug levels of adalimumab in patients with rheumatoid arthritis: an evaluation of costs and effects. Ann Rheum Dis.

[20] Burgos-Vargas R, Tse SM, Horneff G, Pangan AL, Kalabic J, Goss S (2015). A Randomized, Double-Blind, Placebo-Controlled Multicenter Study of Adalimumab in Pediatric Patients With Enthesitis-Related Arthritis. Arthritis Care Res (Hoboken).

[21] Krieckaert C, Hernandez-Breijo B, Gehin JE, le Meledo G, Balsa A, Jani M (2022). Therapeutic drug monitoring of biopharmaceuticals in inflammatory rheumatic and musculoskeletal disease: a systematic literature review informing EULAR points to consider. RMD Open..

[22] Nassar-Sheikh Rashid A, Schonenberg-Meinema D, Bergkamp SC, Bakhlakh S, de Vries A, Rispens T (2021). Therapeutic drug monitoring of anti-TNF drugs: an overview of applicability in daily clinical practice in the era of treatment with biologics in juvenile idiopathic arthritis (JIA). Pediatr Rheumatol Online J.

[23] Verstegen RHJ, McMillan R, Feldman BM, Ito S, Laxer RM (2020). Towards therapeutic drug monitoring of TNF inhibitors for children with juvenile idiopathic arthritis: a scoping review. Rheumatology (Oxford).

[24] Trincianti C, Van Dijkhuizen EHP, Alongi A, Mazzoni M, Swart JF, Nikishina I (2021). Definition and Validation of the American College of Rheumatology 2021 Juvenile Arthritis Disease Activity Score Cutoffs for Disease Activity States in Juvenile Idiopathic Arthritis. Arthritis Rheumatol.

[25] Jabs DA, Nussenblatt RB, Rosenbaum JT, Standardization of Uveitis Nomenclature Working G (2005). Standardization of uveitis nomenclature for reporting clinical data. Results of the First International Workshop. Am J Ophthalmol..

[26] Standardization of Uveitis Nomenclature Working G (2021). Development of Classification Criteria for the Uveitides. Am J Ophthalmol..

[27] Rosas J, Llinares-Tello F, de la Torre I, Santos-Ramirez C, Senabre-Gallego JM, Valor L (2014). Clinical relevance of monitoring serum levels of adalimumab in patients with rheumatoid arthritis in daily practice. Clin Exp Rheumatol.

[28] Chen DY, Chen YM, Tsai WC, Tseng JC, Chen YH, Hsieh CW (2015). Significant associations of antidrug antibody levels with serum drug trough levels and therapeutic response of adalimumab and etanercept treatment in rheumatoid arthritis. Ann Rheum Dis.

[29] Chen DY, Chen YM, Hsieh TY, Hung WT, Hsieh CW, Chen HH (2016). Drug trough levels predict therapeutic responses to dose reduction of adalimumab for rheumatoid arthritis patients during 24 weeks of follow-up. Rheumatology (Oxford).

[30] Papamichael K, Cheifetz AS (2016). Use of anti-TNF drug levels to optimise patient management. Frontline Gastroenterol.

[31] Menting SP, Coussens E, Pouw MF, van den Reek JM, Temmerman L, Boonen H (2015). Developing a Therapeutic Range of Adalimumab Serum Concentrations in Management of Psoriasis: A Step Toward Personalized Treatment. JAMA Dermatol.

[32] Wilkinson N, Tsakok T, Dand N, Bloem K, Duckworth M, Baudry D (2019). Defining the Therapeutic Range for Adalimumab and Predicting Response in Psoriasis: A Multicenter Prospective Observational Cohort Study. J Invest Dermatol.

[33] Samuels A, Whaley KG, Minar P (2023). Precision Dosing of Anti-TNF Therapy in Pediatric Inflammatory Bowel Disease. Curr Gastroenterol Rep..

[34] Franca R, Curci D, Lucafo M, Decorti G, Stocco G (2019). Therapeutic drug monitoring to improve outcome of anti-TNF drugs in pediatric inflammatory bowel disease. Expert Opin Drug Metab Toxicol.

[35] Imagawa T, Takei S, Umebayashi H, Yamaguchi K, Itoh Y, Kawai T (2012). Efficacy, pharmacokinetics, and safety of adalimumab in pediatric patients with juvenile idiopathic arthritis in Japan. Clin Rheumatol.

[36] Kingsbury DJ, Bader-Meunier B, Patel G, Arora V, Kalabic J, Kupper H (2014). Safety, effectiveness, and pharmacokinetics of adalimumab in children with polyarticular juvenile idiopathic arthritis aged 2 to 4 years. Clin Rheumatol.

[37] Hooijberg F, Lami MJ, Berkhout LC, Atiqi S, Nurmohamed M, de Vries A, Krieckaert CL, Rispens T, Wolbink GL (2019). Trough versus non-trough adalimumab drug level measurements. Ann Rheum Dis..

[38] van Herwaarden N BC, van der Maas A, van Vollenhoven RF, Bijlsma JWJ, van den Hoogen FHJ, den Broeder AA, van den Bemt B. Prediction of Successful Dose Reduction or Discontinuation of Adalimumab or Etanercept Using Serum Drug Levels and Antidrug Antibody Measurement: 2014 ACR/ARHP Annual Meeting; 2014 [Available from: https://acrabstracts.org/abstract/prediction-of-successful-dose-reduction-or-discontinuation-of-adalimumab-or-etanercept-using-serum-drug-levels-and-antidrug-antibody-measurement/. 10.1080/17425255.2017.1320390 28425772

[39] Chimenti MS, Triggianese P, Narcisi A, Marinari B, Teoli M, Faleri S (2016). Long-term treatment with adalimumab in psoriatic arthritis: serum adalimumab concentration, immunogenicity and the link with clinical response. J Int Med Res.

[40] Ding X, Zhu R, Wu J, Xue L, Gu M, Miao L (2020). Early Adalimumab and Anti-Adalimumab Antibody Levels for Prediction of Primary Nonresponse in Ankylosing Spondylitis Patients. Clin Transl Sci.

[41] Sanmarti R, Inciarte-Mundo J, Estrada-Alarcon P, Garcia-Manrique M, Narvaez J, Rodriguez-Moreno J (2015). Towards optimal cut-off trough levels of adalimumab and etanercept for a good therapeutic response in rheumatoid arthritis. Results of the INMUNOREMAR study. Ann Rheum Dis..

[42] Hum RM, Ho P, Nair N, Jani M, Morgan AW, Isaacs JD (2022). Non-Trough adalimumab and certolizumab drug levels associated with a therapeutic EULAR response in adherent patients with rheumatoid arthritis.

[43] Ternant D, Bejan-Angoulvant T, Passot C, Mulleman D, Paintaud G (2015). Clinical Pharmacokinetics and Pharmacodynamics of Monoclonal Antibodies Approved to Treat Rheumatoid Arthritis. Clin Pharmacokinet.

[44] Bartelds GM, Wijbrandts CA, Nurmohamed MT, Stapel S, Lems WF, Aarden L (2007). Clinical response to adalimumab: relationship to anti-adalimumab antibodies and serum adalimumab concentrations in rheumatoid arthritis. Ann Rheum Dis.

[45] Vogelzang EH, Kneepkens EL, Nurmohamed MT, van Kuijk AW, Rispens T, Wolbink G (2014). Anti-adalimumab antibodies and adalimumab concentrations in psoriatic arthritis; an association with disease activity at 28 and 52 weeks of follow-up. Ann Rheum Dis.

[46] Skrabl-Baumgartner A, Erwa W, Muntean W, Jahnel J (2015). Anti-adalimumab antibodies in juvenile idiopathic arthritis: frequent association with loss of response. Scand J Rheumatol.

[47] Brunelli JB, Silva CA, Pasoto SG, Saa CGS, Kozu KT, Goldenstein-Schainberg C (2020). Anti-adalimumab antibodies kinetics: an early guide for juvenile idiopathic arthritis (JIA) switching. Clin Rheumatol.

[48] Lovell DJ, Ruperto N, Goodman S, Reiff A, Jung L, Jarosova K (2008). Adalimumab with or without methotrexate in juvenile rheumatoid arthritis. N Engl J Med.

[49] Klein A, Becker I, Minden K, Foeldvari I, Haas JP, Horneff G (2019). Adalimumab versus adalimumab and methotrexate for the treatment of juvenile idiopathic arthritis: long-term data from the German BIKER registry. Scand J Rheumatol.

[50] Lovell DJ, Brunner HI, Reiff AO, Jung L, Jarosova K, Nemcova D (2020). Long-term outcomes in patients with polyarticular juvenile idiopathic arthritis receiving adalimumab with or without methotrexate. RMD Open..

[51] Mahmood I (2016). Pharmacokinetic Considerations in Designing Pediatric Studies of Proteins, Antibodies, and Plasma-Derived Products. Am J Ther.

