# Supplementary material for: Prospective study to characterize adalimumab exposure in pediatric patients with rheumatic diseases
Source: Pediatr Rheumatol Online J. 2024 Jan 2;22:5. doi: 10.1186/s12969-023-00930-8 (PMC10763375; doi:10.1186/s12969-023-00930-8)
Supplement: Supplementary file 1 — Additional file 1: Table S1. Study schedule. Table S2. Sample management. Table S3. JIA subgroups by study group. Table S4 Corticosteroids treatment details at inclusion. Table S5. Grading by cells in the field of the chamber (SUN) and JADAS-10 scores in PRD patients with juvenile idiopathic arthritis and uveitis. Table S6. Univariable and multivariable linear mixed effect models investigating relationship between adalimumab concentrations (log-transformed) and study group, visit age and gender. Table S7. Characteristics adalimumab naïve children receiving the first adalimumab dose. Figure S1. Adalimumab concentrations and inflammatory marker. Figure S2. Adalimumab concentrations and disease activity captured by PGA and PPGA. Figure S3. Adalimumab exposure in children with PRD with adalimumab by study group with and without concomitant corticosteroid treatment. [file 12969_2023_930_MOESM1_ESM.docx]

**Supplemental material**

**Table S1: Study schedule**

|  | **Recruitment** | **Inclusion** | **Naive children (G_N_)** | | **Treatment ≥12 weeks**  **(G_A-M/_G_A_)** | | | **After sample collection** |
| --- | --- | --- | --- | --- | --- | --- | --- | --- |
|  |  |  | **Visit A^#^** | **Visit B^#^** | **First Visit^#^** | **Last Visit^#^** | **Retro-visit*** |  |
| Screening, Informed Consent Process, Coding | x |  |  |  |  |  |  |  |
| Collection of baseline information (e.g. gender, age at diagnosis, diagnosis) |  | x |  |  |  |  |  |  |
| Vital signs, bodyweight, height |  |  | x | x | x | x | x |  |
| Screening for infection |  |  | x | x | x | x |  |  |
| Patients History (e.g. morning stiffness, pain) |  |  | x |  | x | x | (x) |  |
| Physical examination and active joint count |  |  | x |  | x | x | (x) |  |
| Uveitis assessment (SUN grade) |  |  | x |  | x | x | (x) |  |
| Physician global assessment (PGA; VAS 0-10cm) |  |  | x |  | x | x | (x) |  |
| Patients/parents global assessment (PPGA; VAS 0-10 cm) |  |  | x |  | x | x | (x) |  |
| JIA: Disease activity assessment (JADAS-10) |  |  | x |  | x | x | x |  |
| Assessment of treatment regimen adalimumab +/.- methotrexate |  |  | x | x | x | x | x |  |
| Assessment concomitant treatment |  |  | x | x | x | x | x |  |
| Laboratory routine parameters^1^ |  |  |  |  | x | x | x |  |
| Adalimumab sample^2^ |  |  | x |  |  |  |  |  |
| Adalimumab sample^3^ |  |  |  | x |  |  |  |  |
| Adalimumab sample^4^ |  |  |  |  | x | x |  |  |
| Sample management^5^ |  |  | x | x | x | x |  |  |
| Butch analysis of adalimumab samples |  |  |  |  |  |  |  | x |

**Legend**: **^#^** prospective data collection, *retrospective data collection for all performed visits between first visit and adalimumab treatment start, X data collected, (X) data collection if available in e-health record,^1^ Laboratory routine parameter (e.g. whole blood count, C-reactive protein, erythrocyte sedimentation rate), ^2^ 3 to 7 days after first adalimumab administration, ^3^ 10 to 14 days after first adalimumab administration, ^4^ C_max_ = 1 to 9 days after adalimumab administration, C_min_ =10 to 14 days after adalimumab administrations, ^5^ please refer to supplemental material S2. Abbreviation: *VAS* Visual analogue scale (0 representing no disease activity; 10 representing maximum disease activity); *JADAS* Juvenile Arthritis Disease Activity Score; *SUN* standardization of the uveitis nomenclature.

**Table S2. Sample management**

| **Steps** | **Task** | **Description** |
| --- | --- | --- |
| 1 | PK sampling | 1.2 mL full blood in serum tubes (blood draw according to hospital standard operation procedures) |
| 2 | Centrifugation | 30 minutes after PK sampling (2500 g, 10 minutes, 20°Celsius) |
| 3 | Aliquots | Standardized tubes (aliquots of 0.5 mL) |
| 4 | Aliquot labeling | Standardized barcodes (linked with subject ID in line with the subject enrollment log) |
| 5 | Freezing | -80°Celsius degree (fridge with temperature log) for ≤ 11 months |
| 6 | Sample storage log | Subject-ID, aliquot code, aliquot storage position, sample collection and freezing datetime |
| 7 | Transport | On dry ice |
| 8 | Analysis | MVZ Dr Eberhard & Partner Dortmund, Dortmund, Germany |

**Table S3. JIA subgroups by study group**

|  | **Study group A-M (G_A-M_)**  **(n= 14)** | **Study group A (G_A_)**  **(n=14)** |
| --- | --- | --- |
| **Oligoarticular JIA** | 7 (50%) | 4 (28.6%) |
| - With uveitis | 6 | 3 |
| - Without uveitis | 1 | 1 |
| **Polyarticular JIA, RF-** | 2 (14.3%) | 2 (14.3%) |
| **Polyarticular JIA, RF+** | 1 (7.1%) | 0 |
| **ERA** | 2 (14.3%) | 2 (14.3%) |

Abbreviation: *G_A-M_* study group adalimumab and methotrexate, *G_A_* study group adalimumab, *JIA* juvenile idiopathic arthritis; *RF* rheumatoid factor + positive, - negative, *ERA* Enthesitis related arthritis

**Table S4 Corticosteroids treatment details at inclusion**

| Pat | Diagnosis | Ocular steroids | | Systemic corticosteroids | | |
| --- | --- | --- | --- | --- | --- | --- |
|  |  | Drops/Eye | Frequency | Dose (mg/kg) | Route | Frequency |
| 1 | OJIA with uveitis | 1 (both eyes) | q2d | No | | |
| 2 | Idiopathic uveitis | No | | 0.09^1^ | p.o | qd |
| 3 | OJIA with uveitis | 1 (both eyes) | qd | No | | |
| 4 | OJIA with uveitis | 1 (left eye) | q2d | No | | |
| 5 | Idiopathic uveitis | 1 (right eye) | qd | No | | |
| 6 | PJIA | No | | 0.08^2^ | p.o | qd |

Note: One patient with OJIA and uveitis started ocular corticosteroids with 1 drop/eye daily after study inclusion.

Abbreviations: OJIA Oligoarticular juvenile idiopathic arthritis, PJIA Polyarticular juvenile idiopathic arthritis, qd daily, q2d every second day, ^1^ Prednisone, ^2^ Hydrocortisone.

|  | | **Study group A-M (G_A-M_)** | | | | **Study group A (G_A_)** | | | |
| --- | --- | --- | --- | --- | --- | --- | --- | --- | --- |
|  |  | Idiopathic Uveitis, n=2 | OJIA, n=7  (n= 6 with Uveitis) | PJIA, n=3 | ERA, n=2 | Idiopathic Uveitis, n=5 | OJIA, n=4  (n= 3 with Uveitis) | PJIA, n=2 | ERA, n=2 |
| **First study visit** | | | | | | | | | |
| SUN, n (%) | 0 | 2 (100) | 6 (100) | n.a. | n.a. | 4 (80) | 1 (33.3) | n.a. | n.a. |
|  | 0.5+ | 0 | 0 |  |  | 1 (20) | 1 (33.3) |  |  |
|  | 1+ | 0 | 0 |  |  | 0 | 0 |  |  |
|  | 2+ | 0 | 0 |  |  | 0 | 1 (33.3) |  |  |
| JADAS-10 | Median, [IQR] | n.a. | 1.00 [0.25, 1.75] | 6.00 [5.5, 10.0] | 4.00 [4.0, 4.0] | n.a. | 1.0 [0.75, 2.0] | 1.5 [0.75, 2.25] | 0 [0, 0] |
|  | Range (min, max) |  | 0, 3.0 | 5.0, 14.0 | 4.0, 4.0 |  | 0, 5.0 | 0, 3.0 | 0, 0 |
| **Last study visit** | | | | | | | | | |
| SUN, n (%) | 0 | 2 (100) | 3 (50) | n.a. | n.a. | 5 (100) | 2 (66.7) | n.a. | n.a. |
|  | 0.5+ | 0 | 2 (33.3) |  |  | 0 | 0 |  |  |
|  | 1+ | 0 | 0 |  |  | 0 | 1 (33.3) |  |  |
|  | 2+ | 0 | 0 |  |  | 0 | 0 |  |  |
|  | Missing | 0 | 1 (16.7) |  |  | 0 | 0 |  |  |
| JADAS-10 | Median, [IQR] | n.a. | 1.00 [0.5, 3.0] | 6.00 [5.0, 7.0] | 4.50 [3.75, 5.25] | n.a. | 0 [0, 1.5] | 0 [0, 0] | 0 [0, 0] |
|  | Range (min, max) |  | 0, 4.0 | 4.0, 8.0 | 3.0, 6.0 |  | 0, 6.0 | 0, 0 | 0, 0 |

**Table S5. Grading by cells in the field of the chamber (SUN) and JADAS-10 scores in PRD patients with juvenile idiopathic arthritis and uveitis**

Abbreviation: *G_A-M_* study group adalimumab and methotrexate, *G_A_* study group adalimumab, *OJIA* Oligoarticular juvenile idiopathic arthritis, *PJIA* Polyarticular juvenile idiopathic arthritis*, ERA* Enthesitis related arthritis; standardization of the uveitis (SUN) nomenclature; *JADAS* Juvenile Arthritis Disease Activity Score, n.a. not applicable

**Table S6. Univariable and multivariable linear mixed effect models investigating relationship between adalimumab concentrations (log-transformed) and study group, visit age and gender**


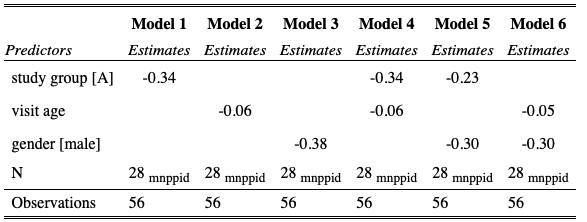


Abbreviation: A study group adalimumab, A-M study group adalimumab and methotrexate, NA not applicable. Interactions were also fitted, but were not significant at the 5% level.

**Table S7. Characteristics adalimumab naïve children receiving the first adalimumab dose**

|  | **Adalimumab naive patients receiving first dose (n=8)** |
| --- | --- |
| **Sex, n (%)** |  |
| Female | 6 (75) |
| **Median age, years [IQR]** |  |
| At diagnosis | 12.8 [10.5, 15.0] |
| At Visit A | 12.8 [11.0, 15.6] |
| **Biometric information** |  |
| Median body weight, kg [IQR] | 45.8 [43.2, 56.7] |
| Body weight < 30 kg, n (%) | 1 (12.5) |
| Median BSA, m^2^ [IQR] | 1.41 [1.33, 1.59] |
| **Diagnosis, n (%)** |  |
| JIA | 5 (62.5) |
| Idiopathic Uveitis | 3 (37.5) |
| CRMO | 0 |
| Comorbidities | 0 |
| **Adalimumab treatment, median [IQR]** |  |
| Dose absolute, mg | 40.0 [40.0, 40.0] |
| Dose per BSA, m^2^ | 28.4 [25.2, 30.0] |
| Administration frequency, days | 14.0 [14.0, 14.0] |
| **Methotrexate treatment** |  |
| Patients with concomitant Methotrexate, n (%) | 5 (62.5) |
| Median time since start, months [IQR] | 2.33 [1.51, 3.98] |
| Median dose absolute, mg [IQR] | 15.0 [15.0, 15.0] |
| Median dose per BSA, m^2^ [IQR] | 9.62 [8.93, 10.6] |
| Median administration frequency, days [IQR] | 7.00 [7.00, 7.00] |
| **Adalimumab concentration, median** [IQR] |  |
| C_max_ (3-7 days) | 7.10 [4.63, 8.60] |
| C_trough_ (10-14 days) | 5.80 [3.80, 7.25] |

**Abbreviation:** *BSA* Body surface area, *CRMO* chronic recurrent multifocal osteomyelitis, *IQR* Inter-quartile ranges, *i.v.* intravenous*, JIA* juvenile idiopathic arthritis, *kg* kilogram, *mg* milligram, *n.a.* not available, *NSAIDS* non-steroidal anti-inflammatory drugs, *p.o* per os

**Figures**

**S1 Adalimumab concentrations and inflammatory marker**

**
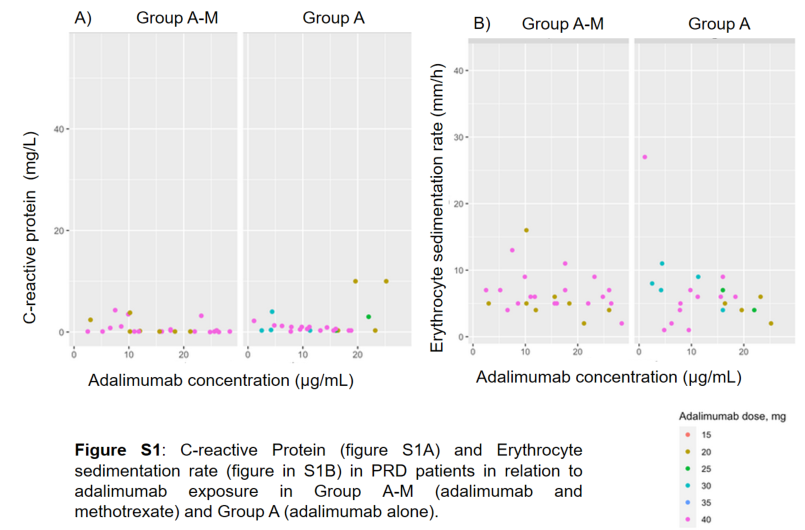
**

**S2 Adalimumab concentrations and disease activity captured by PGA and PPGA**


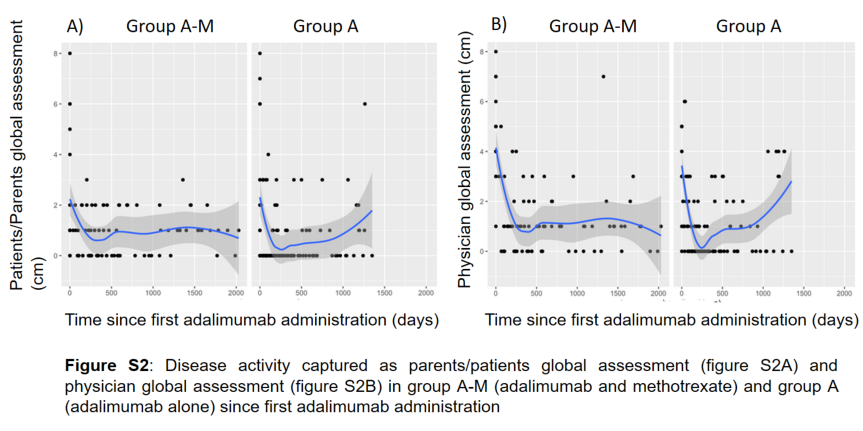


**S3 Adalimumab exposure in children with PRD with adalimumab by study group with and without concomitant corticosteroid treatment.**

**
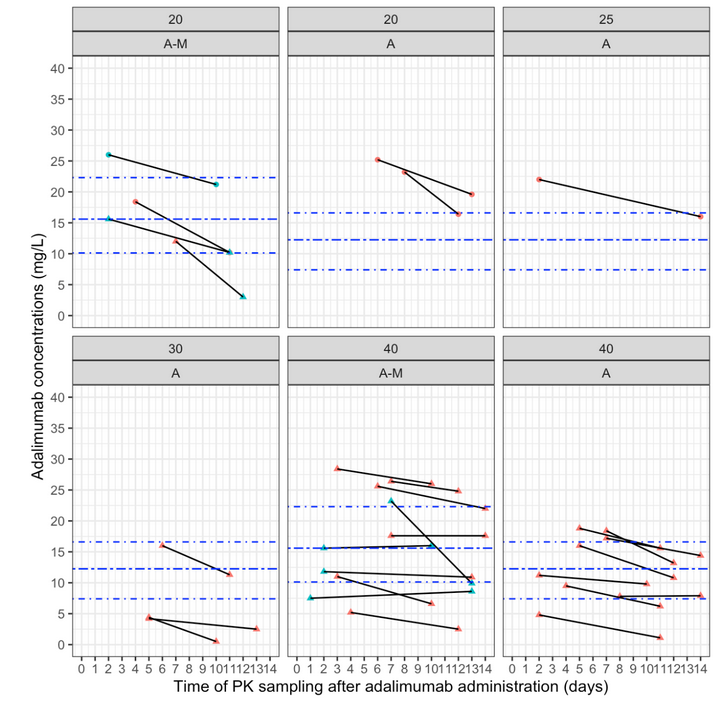
**

Legend: Adalimumab exposure in children with PRD treated with adalimumab and methotrexate (A-M) or adalimumab alone (A) ≥12 weeks with adalimumab absolute doses of 20, 25, 30 or 40 mg with or without corticosteroid administration. Maximum adalimumab concentrations were collected after 1 to 9 days (C_max_) and minimum concentrations after 10 to 14 days (C_min_). The *dash blue lines* represent the interquartile ranges [IQR] and the median concentrations per study group (A-M: 15.6 mg/L [IQR 10.1, 22.3]; A: 12.3 mg/L [IQR 7.4, 16.6]). *Triangle*: body weight ≥30 kg, *dot*: body weight <30 kg. *Blue coloured dot/triangle*: ocular steroids or systemic corticosteroids. *Red coloured dot/triangle*: no ocular steroids or systemic corticosteroids.
